# Supplementary material for: Nitrogen-Doped Porous MXene (Ti3C2) for Flexible Supercapacitors with Enhanced Storage Performance
Source: Molecules. 2022 Jul 30;27(15):4890. doi: 10.3390/molecules27154890 (PMC9369756; doi:10.3390/molecules27154890)
Supplement: Supplementary file 1 [file molecules-27-04890-s001.zip › molecules-1807288-supplementary.pdf]

molecules

Supplementary Materials

Nitrogen-Doped Porous MXene (Ti_3C_2) for Flexible Supercapacitors with Enhanced Storage Performance

Xin Tao [1,2,†], Linlin Zhang [1,2,†], Xuedong He [3], Lingzi Fang [2], Hongyan Wang [2], Li Zhang [2], Lianghao Yu [2,*] and Guang Zhu [2,*]

[1] School of Mechanics and Optoelectronic Physics, Anhui University of Science and Technology, Huainan 232001, China; taox5802311013@163.com (X.T.); 17805572596@163.com (L.Z.)
[2] Key Laboratory of Spin Electron and Nanomaterials of Anhui Higher Education Institutes, School of Mechanical and Electronic Engineering, Suzhou University, Suzhou 234000, China; fanglz0@163.com (L.F.); suzhouwhy@163.com (H.W.); zhlisuzh@163.com (L.Z.)
[3] Key Laboratory of Leather of Zhejiang Province, College of Chemistry and Materials Engineering, College of Chemistry & Materials Enginnering, Wenzhou University, Wenzhou 325035, China; xuedonghe7878@gmail.com
* Correspondence: lhyu@ahszu.edu.cn (L.Y.); guangzhu@ahszu.edu.cn (G.Z.)
† These authors contributed equally to this work.

Electrochemistry

All electrochemical properties were measured on the CHI 660E electrochemical workstation. For the three-electrode test systems, the active material was a working electrode, Ag/AgCl in saturated KCl was a reference electrode, and platinum wire was a counter electrode. To make the working electrode, the electroactive material, conductive carbon black, and polyvinylidene fluoride (PVDF) were stirred vigorously in N-Methylpyrrolidone (NMP), in the weight ratio of 70:20:10. The slurry was uniformly coated on a 1 cm × 1 cm titanium mesh substrate and dried overnight at 60 °C in a vacuum for 12 h. The weight of each electrode was about 1 mg. CV and GCD were tested in 2 M H_2SO_4 electrolyte solutions, by scanning the potential range (-0.2-0.4 V) at scan rates of 1-100 mV s^{-1} and different current densities of 1-10 A g^{-1}. An EIS test was over a frequency range of 0.01 Hz to 100 kHz.

Supercapacitor Device

Two-piece same-quality N-MXene-F is directly pressed on two titanium mesh to assemble a symmetrical supercapacitor (SSC), isolating the two electrodes separated by a glass fiber diaphragm and soaking in a 2 M H_2SO_4 electrolyte.

The energy density and power density of the SSC were calculated as follows:

$$E = \frac{1}{2}\mathrm{C}(\Delta \mathrm{V})^2 \tag{1}$$

$$P = \frac{\mathrm{E} \times 3600}{\Delta \mathrm{t}} \tag{2}$$

where E represents the energy density, C is the specific capacitance, P represents the power density, ΔV is the operating voltage window, and Δt is the discharge time.

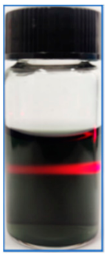

Figure S1. Digital photo of MXene dispersion with obvious Tyndall effect.

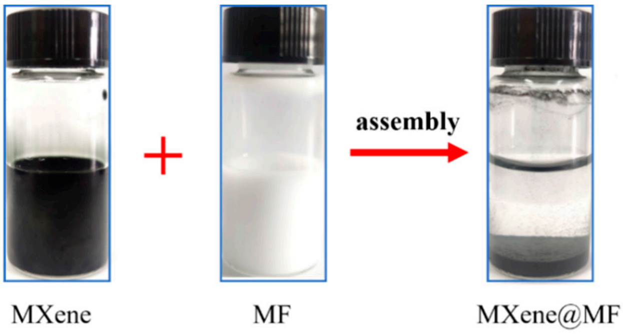

Figure S2. MXene and MF occur in an electrostatic self-assembly process.

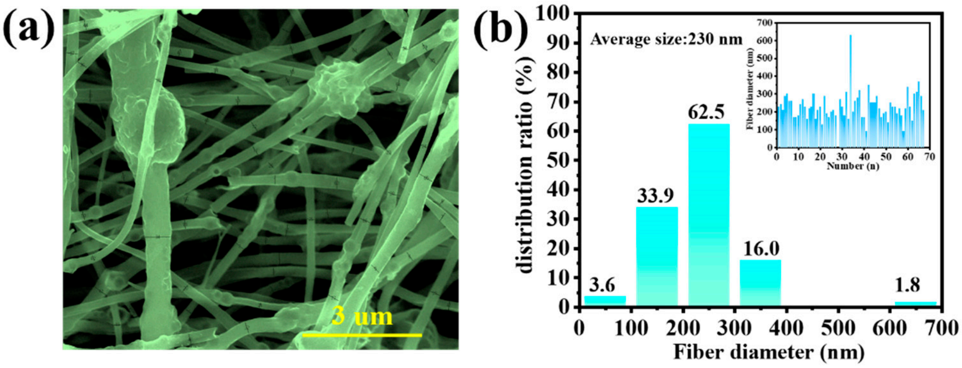

Figure S3. (**a**) SEM image of N-MXene-F. (**b**) Fiber-diameter distribution.

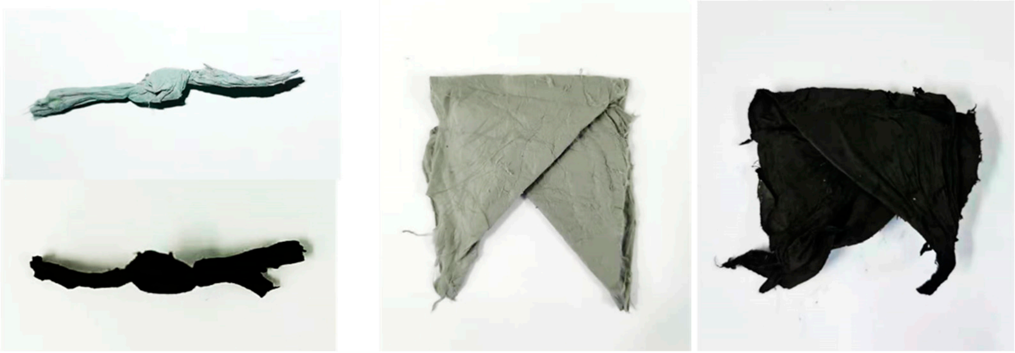

Figure S4. Digital photos of N-MXene-F folded into various shapes.

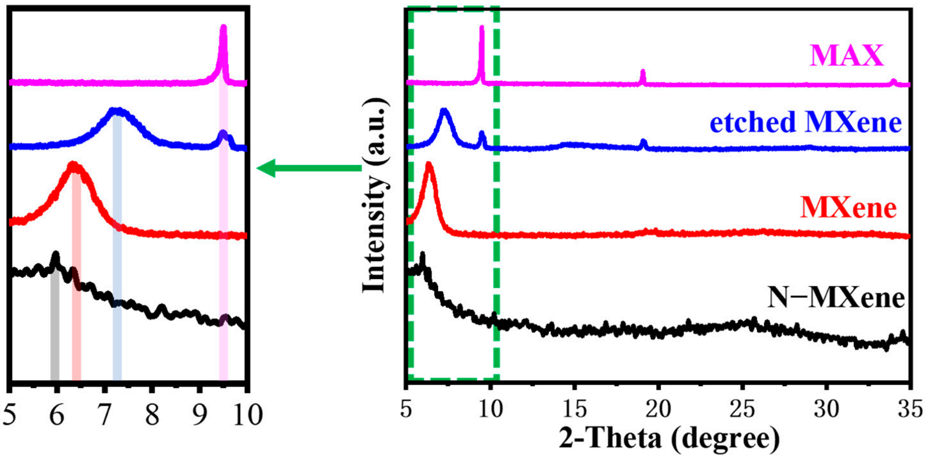

Figure S5. XRD patterns of MAX, etched MXene, exfoliated MXene, and N-MXene.

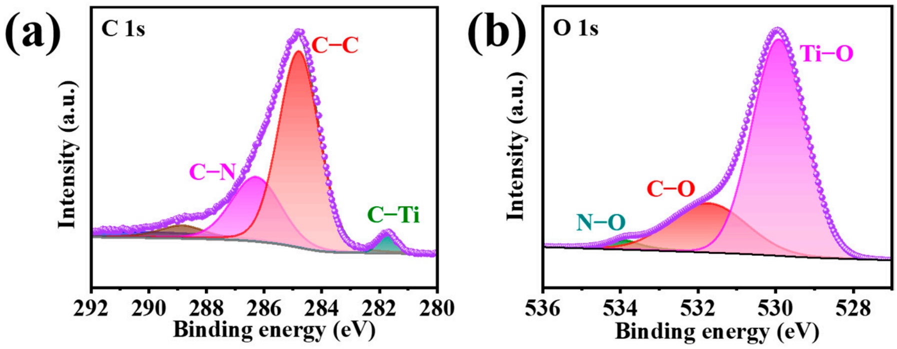

Figure S6. High-resolution (**a**) C 1s and (**b**) O1s spectra of N-MXene.

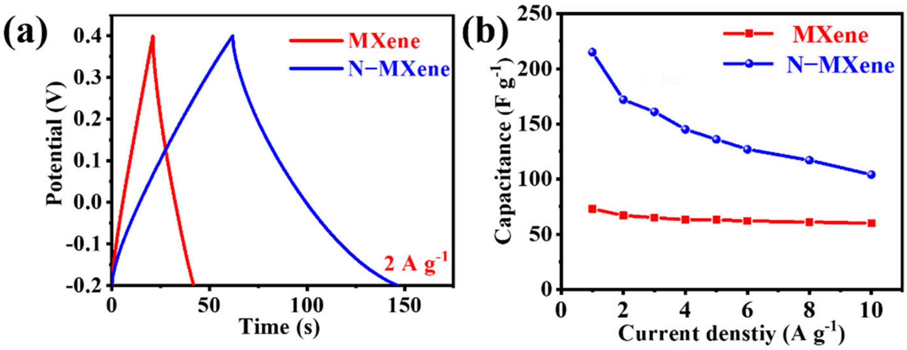

Figure S7. Electrochemical performance of the three-electrode system of MXene and N-MXene electrodes: (**a**) GCD curves; (**b**) specific capacitance at different current densities.

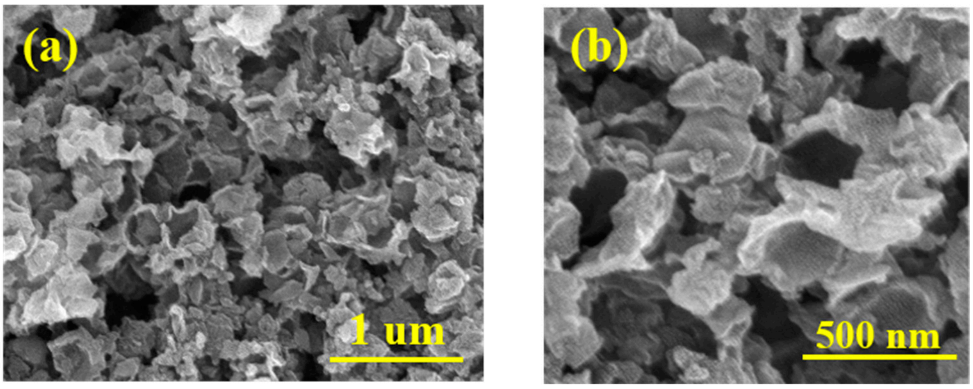

Figure S8. (**a**) SEM of N-MXene electrode before cycling; (**b**) after cycling.

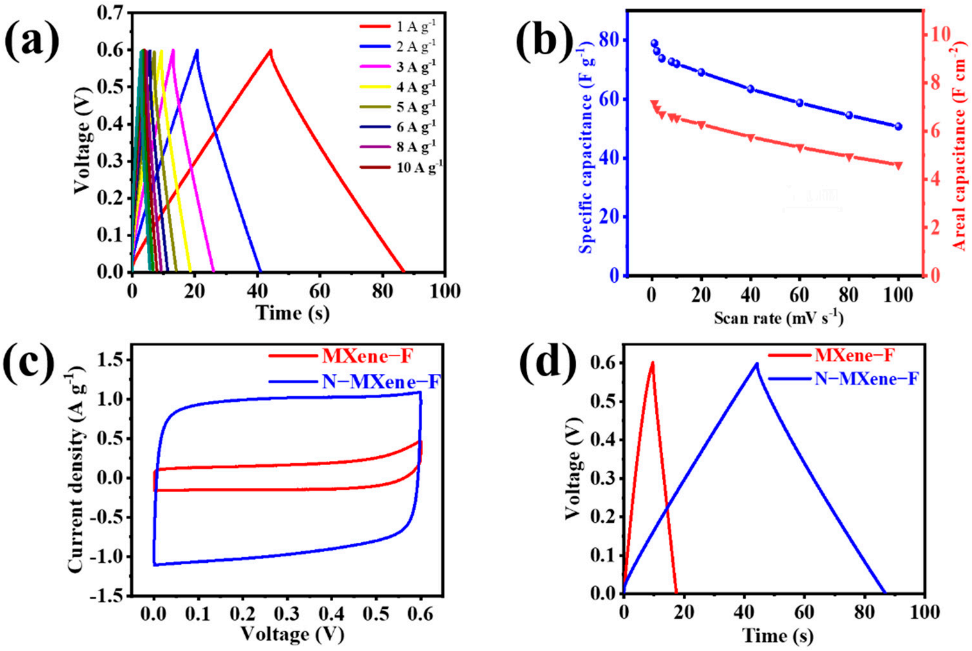

Figure S9. (**a**) Electrochemical properties of MXene-F and N-MXene-F-based SSC: GCD curves of N-MXene-F-based SSC at different current densities; (**b**) specific and areal capacitance of N-MXene-F-based SSC at different scan rates; (**c**) CV curve at a scan rate of 5 mV s^{-1}; (**d**) GCD curve at a current density of 1 A g^{-1}.

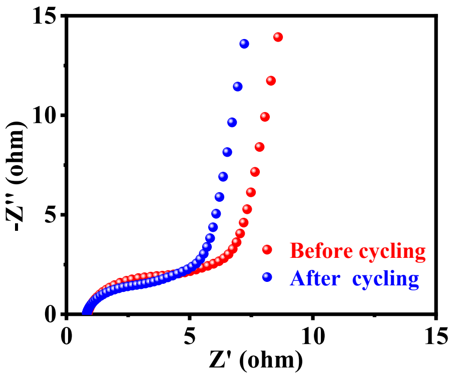

Figure S10. Nyquist plots of the N-MXene-F-based SSC before and after cycles.

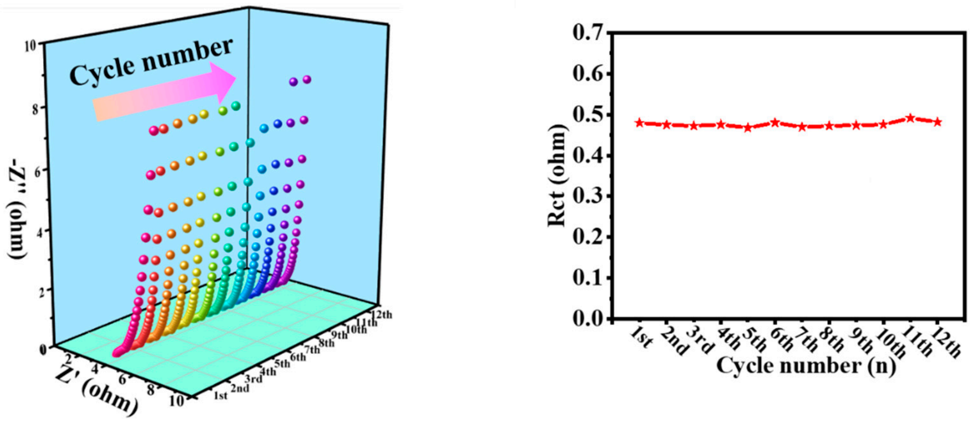

Figure S11. (**a**) EIS of N-MXene-F-based SSC at different bending times; (**b**) corresponding Rct.

Table S1. The atomic concentration of elements detected from XPS measurement for N-MXene.

Element	Content (%)
C1s	56.97
N1s	10.37
Ti2p	12.21
O1S	20.44
